# Induced pluripotent stem cell-derived and directly reprogrammed neurons to study neurodegenerative diseases: The impact of aging signatures

**DOI:** 10.3389/fnagi.2022.1069482

**Published:** 2022-12-20

**Authors:** Simona Aversano, Carmen Caiazza, Massimiliano Caiazzo

**Affiliations:** ^1^Department of Molecular Medicine and Medical Biotechnology, University of Naples Federico II, Naples, Italy; ^2^Department of Pharmaceutics, Utrecht Institute for Pharmaceutical Sciences (UIPS), Utrecht University, Utrecht, Netherlands

**Keywords:** cell reprogramming, Parkinson’ disease, Alzheimer’s disease, Huntington’s disease, ALS, *in vitro* model

## Abstract

Many diseases of the central nervous system are age-associated and do not directly result from genetic mutations. These include late-onset neurodegenerative diseases (NDDs), which represent a challenge for biomedical research and drug development due to the impossibility to access to viable human brain specimens. Advancements in reprogramming technologies have allowed to obtain neurons from induced pluripotent stem cells (iPSCs) or directly from somatic cells (iNs), leading to the generation of better models to understand the molecular mechanisms and design of new drugs. Nevertheless, iPSC technology faces some limitations due to reprogramming-associated cellular rejuvenation which resets the aging hallmarks of donor cells. Given the prominent role of aging for the development and manifestation of late-onset NDDs, this suggests that this approach is not the most suitable to accurately model age-related diseases. Direct neuronal reprogramming, by which a neuron is formed *via* direct conversion from a somatic cell without going through a pluripotent intermediate stage, allows the possibility to generate patient-derived neurons that maintain aging and epigenetic signatures of the donor. This aspect may be advantageous for investigating the role of aging in neurodegeneration and for finely dissecting underlying pathological mechanisms. Here, we will compare iPSC and iN models as regards the aging status and explore how this difference is reported to affect the phenotype of NDD *in vitro* models.

## Introduction

Aging is the natural process that progressively leads to the functional decline of the cell and ultimately of the whole organism. While its nature and causes still belong to a debated field, researchers mainly addressed the non-trivial question of defining aging at a biological level. Biological aging is very complex and involves genomic mutations, epigenetic changes, telomere shortening, and progressive decline in metabolic function and proteostasis, all reflecting different aspects of the process (Gladyshev, 2016). The aging signatures are crucial to correctly establish cellular age independently of chronological age and understand the impact of each factor on physiological and pathological aspects of aging itself. In this regard, aging has emerged as the leading risk factor for the late-onset diseases and particularly in neurodegenerative diseases (NDDs; Gudenschwager et al., 2021), thereby posing the urge to unveil its contribution in NDDs development. NDDs comprise a group of disorders that affect central nervous system (CNS) and have a higher incidence among the aged population. Among them, the most studied are Alzheimer’s disease (AD), Parkinson’s disease (PD), amyotrophic lateral sclerosis (ALS), and Huntington’s disease (HD).

Although the genes involved in some early-onset familiar forms of the diseases were identified, the overwhelming proportion of sporadic diagnosed cases remains unexplained (Wang et al., 2008; Fernández-Santiago et al., 2015) and effective treatment strategies to cure these diseases are missing. Aging hallmarks could account for the late-onset forms of NDDs, presumably interacting with genetic and environmental factors. Finding appropriate disease models that include aging information may help to achieve a comprehensive understanding of disease mechanisms and progression and to identify potential therapeutic targets.

Recently, the possibility of studying adult human neurons obtained by cell reprogramming strategies provided precious insights into the molecular mechanisms underlying neurodegeneration and other neurological disorders. Neurons can be derived from induced pluripotent stem cells (iPSCs) or directly induced from somatic cells such fibroblasts (iNs) to make patient-tailored disease models. In the first strategy, primary human cells can be collected directly from patients, and the cells can then be reset to an embryonic-like state with the transient expression of the Yamanaka transcription factors (TFs), OCT4, KLF4, SOX2, and C-MYC (OKSM; Takahashi and Yamanaka, 2006). The generated iPSCs have the potential to differentiate into all three germ layers with the unlimited ability of self-renewal, providing a potential source of functional neurons that can be obtained either by using several developmental morphogens and small molecules (Kriks et al., 2011) or by forced expression of few neurogenic TFs. In the second case it is possible to apply forced expression of TFs directly to iPSCs to generate in a faster way (~3 weeks) dopamine, glutamate, GABA, and motor neurons (Dimos et al., 2008; Chambers et al., 2009; Maroof et al., 2013; Theka et al., 2013; Chen et al., 2014; Yang et al., 2017).

The alternative route to obtain mature and functional neurons is the direct reprogramming of somatic cells into neurons, over-expressing combinations of neurogenic TFs. The first described example of this approach was the generation of glutamate neurons by applying ASCL1, BRN2, and MYT1L (BAM factors) on mouse embryonic fibroblasts (Vierbuchen et al., 2010).

Both strategies have also been used to obtain specific neural subtypes that are relevant for NDDs modeling; nevertheless, they can recapitulate different phenotypic properties of diseases. The differential maintenance of aging status of donor cells is the reason for this discrepancy and an aspect that has come to the attention of the researchers aiming to faithfully model NDDs.

Essentially, the reprogramming tools follow a different developmental program toward the neural fate. The induction of pluripotency entails an embryonic-like state which resets the cellular age and leads to cell rejuvenation (Lapasset et al., 2011) even in the finally differentiated neurons (Huh et al., 2016). Direct conversion of fibroblasts into neurons circumvents intermediate embryonic cell stages and has proven to preserve the cellular age of donor cells (Huh et al., 2016). Obligatory access to patient cell lines is a limitation of these approaches, since NDDs modeling needs the generation of iPSCs or iNs from large cohorts of patients, to comprise the genetic heterogeneity with respect to the disease-causing mutations. This limitation has been expunged by CRISPR/Cas9-edited disease models, which allow to introduce or correct any desired mutation, and to obtain isogenic cell lines carrying one or multiple disease-relevant mutations from a single iPSC background (Sen and Thummer, 2022). In this review, we describe in more detail the differences in the aging status of iN- and iPSC-derived neurons and discuss the impact of these features in the disease modeling of NDDs.

## Aging features in directly converted and iPSC-derived neurons

### Epigenetic and transcriptional memory

**Figure 1:**
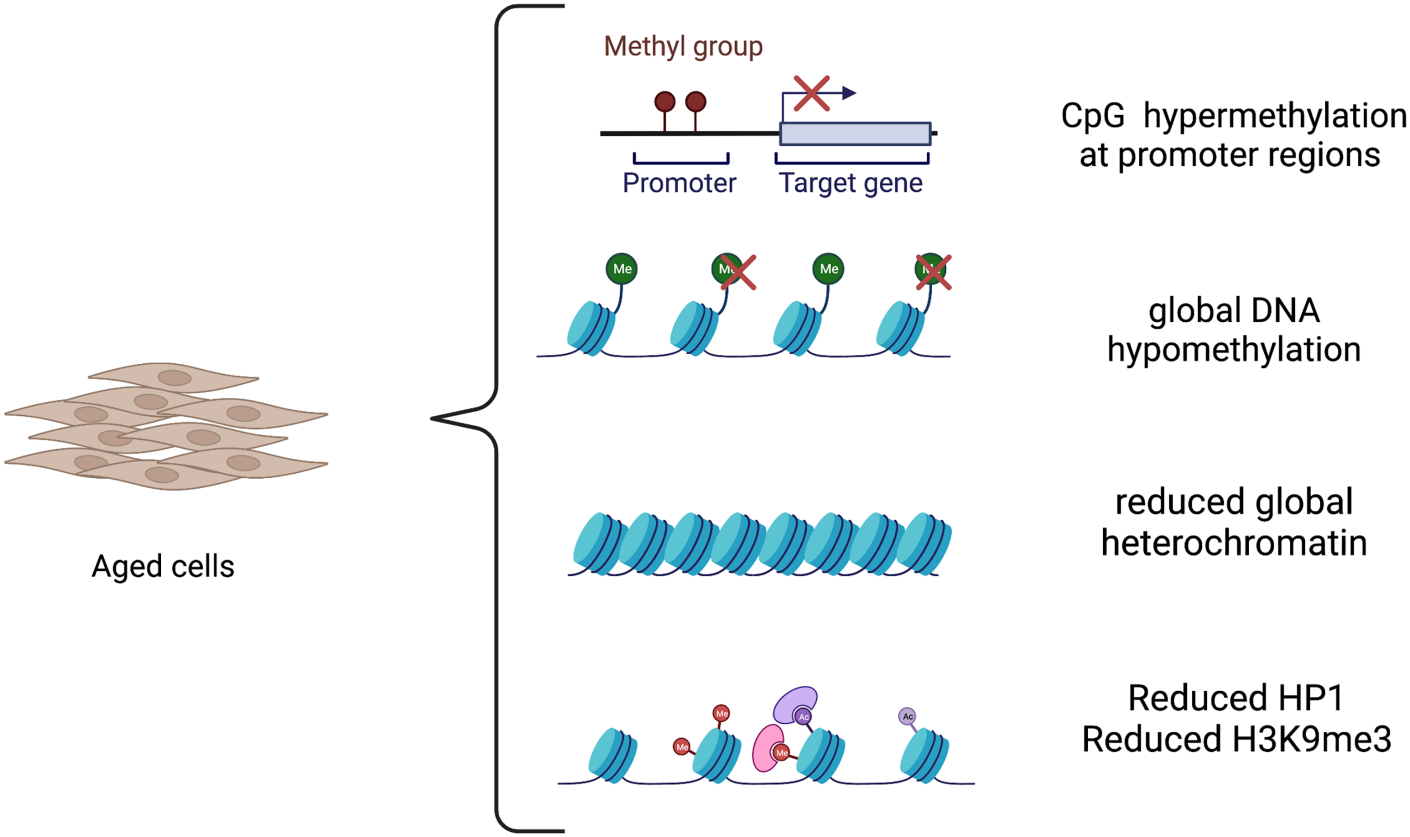
The epigenetic landscape in aging. Aging causes both global and locus-specific changes to chromatin structure. The aging process is characterized by global DNA hypomethylation except in CpG islands that become hypermethylated at promoter regions of tissue-specific genes. Moreover, there is a decrease in global heterochromatin along with a reduction in heterochromatin protein 1 (HP1) and in repressive histone mark H3K9me3.

Age-associated changes in TFs and chromatin state represent a critical aspect of the aging process and a link between different aging hallmarks. Aging changes include reduced global heterochromatin, nucleosome loss and remodeling, changes in histone marks, global DNA hypomethylation with CpG island hypermethylation, and the relocalization of chromatin modifying factors (Oberdoerffer and Sinclair, 2007; Campisi and Vijg, 2009). Importantly, these factors were demonstrated to be correlated with age-related neurodegeneration (Booth and Brunet, 2016), albeit their contribution for disease development is far to be dissected. An *in vitro* model that reliably retains the epigenetic information is thus required to achieve a full comprehension of the sporadic as well genetic forms of the diseases. The epigenetic landscape in aging is depicted in Figure 1.

The altered methylation landscape has been calibrated to accurately predict the age of cells (Horvath, 2013) and allowed for accurate aging characterization of iPSC-derived neurons and iNs, shedding light onto the epigenetic mechanisms governing the two different reprogramming strategies and the effects on the maintenance of cellular age. The induction of pluripotency resets the epigenetic clock to zero (Horvath and Raj, 2018) confirming previous evidence that demonstrated cell rejuvenation by reprogramming through the pluripotent state (Maherali et al., 2007; Meissner et al., 2008; Lapasset et al., 2011). Of note, epigenetic age is not restored upon neural induction, independently from the age of donor cells (Huh et al., 2016). This aspect can be explained by the hierarchical transcriptional and epigenetic mechanisms that occur during the TF-mediated induction of the pluripotent state. The reprogramming process involves the “off-target” cooperative chromatin binding of TFs, the OKSM factors identified by Yamanaka’s group (Takahashi and Yamanaka, 2006). TFs need to overcome a series of epigenetic barriers that have been gradually imposed on the genome during differentiation, including large, repressed chromatin domains enriched by H3K9me3 modification, to induce a huge chromatin remodeling that finally leads to the erasure of somatic acquired epigenetic marks and reactivation of pluripotent genes (Takahashi and Yamanaka, 2006; Takahashi et al., 2007; Apostolou and Hochedlinger, 2013; Wapinski et al., 2013). It has been shown that age reset precedes de-differentiation during iPSC reprogramming with OSKM factors (Olova et al., 2019; Gill et al., 2022) and that combinations of two or three of those factors lead to similar rejuvenation effects, even after 2–3 days of exposure, where aging signatures are partially erased and restored to youthful states (Lu et al., 2020).

As expected, the epigenetic rejuvenation of iPSCs results in a population of reprogrammed neural cells that are epigenetically and transcriptionally different from their *in vivo* counterpart, hampering their potentiality for *in vitro* modeling and cell replacement therapy.

A genome-wide comparative gene-expression profiling demonstrated a clear correlation between iPSC-derived mesencephalic dopaminergic (mDA) neurons and embryonic mDA neurons, but less similarity was found between iPSC-derived mDA neurons and postnatal mDA neurons (Roessler et al., 2014). In stark contrast to iPSC-derived neurons, direct neuronal conversion from fibroblasts sampled at different ages has proven to retain the epigenetic age of the original donor cells (Huh et al., 2016). iNs do not follow the precise intermediate states of development but generate a unique intermediate state that is unrelated to the donor and the target cells. Despite this ‘shortcut’, iNs appear to arrive at the same state as neurons obtained by differentiation, preserving epigenetic information about age and disease (Traxler et al., 2019). It is worth noting that direct neural conversion is dependent on the activity of “on-target” pioneer TFs which immediately bind pro-neural target genes such as ASCL1 that facilitates the further action of BRN2 and MYT1L (BAM factors; Wapinski et al., 2013; Smith et al., 2016) and that these events do not erase the somatic epigenetic marks to induce an intermediate pluripotent cell stage. Therefore, direct reprogramming appears as the most suitable *in vitro* tool to obtain patient-specific neurons that also include the epigenetic information.

The concept of epigenetic memory was previously applied to iPSCs and neurons differentiated from iPSCs. These studies showed that iPSCs retain the epigenetic signatures of donor cells, which also predispose them to a preferential lineage-specific differentiation (Bar-Nur et al., 2011; Kim K. et al., 2011). DA neurons derived from iPSCs are somehow different from their primary counterpart, since they show a differential expression relative to fibroblast specific markers, suggesting remnants of a still active fibroblast gene program in iPSC-derived DA neurons (Roessler et al., 2014). However, these data were not conclusive and have to be read in the light of other studies showing that embryonic stem cells (ESCs) themselves exhibit variation in terms of their growth profiles and differentiation potential (Osafune et al., 2008; Sullivan et al., 2010). Finally, both iPSCs and ESCs share pluripotency and self- renewal capacity, indicating that transcriptional or epigenetic differences have no overall significant impact on stem-cell pluripotency *per se* nor are directly correlated to epigenetic memory. Instead, these subtle differences may well be compounded by culture conditions and laboratory practices (Sullivan et al., 2010).

Apart from the classical CpG DNA methylation which has an established role in gene regulation, some studies have underpinned the relevance of non-CpG methylation in the epigenetic landscape of adult mammalian brains, where mCH (where H = A, T, or C) and intermediates in the DNA demethylation pathway, in particular 5-hydroxymethylcytosine (5hmC; Xie et al., 2012; Guo et al., 2014; He and Ecker, 2015), are found at high levels.

The precise role of these modifications is not fully understood; however, several evidence reported that mCH takes part in the modulation of gene expression in mature neurons (Xie et al., 2012; Mo et al., 2015; Stroud et al., 2017). mCH starts with birth and is targeted to constitutive repressed genes and to genes showing developmental downregulation in mature neurons (Luo et al., 2019), suggesting that this mechanism facilitates neuronal maturation through gene repression. Therefore, mCH represents an essential component of the epigenetic landscape of mature neurons, which has a profound implication in iPSC- and iN-based disease models. Whole-genome profiles of DNA methylation in iPSCs showed an aberrant methylation in the non-CpG context which is later transmitted to differentiated cells (Lister et al., 2011). Instead, base-resolution methylome of fully differentiated mouse iNs showed consistent mCH patterns; however, they did not recapitulate perfectly mCH and showed a different promoter CpG methylation compared to mature cortical neurons (Luo et al., 2019). This is in agreement with a recent investigation in which neurons differentiated from mouse ESCs have proven to acquire CH methylation in a similar time frame as their *in vivo* counterparts, but they still do not recapitulate this methylation context with fidelity (Martin et al., 2020). These data suggest that other factors are necessary to fully develop *in vivo* methylation levels and patterns and this aspect must be deepened in order to faithfully recapitulate the aged neuronal methylome.

**Figure 2:**
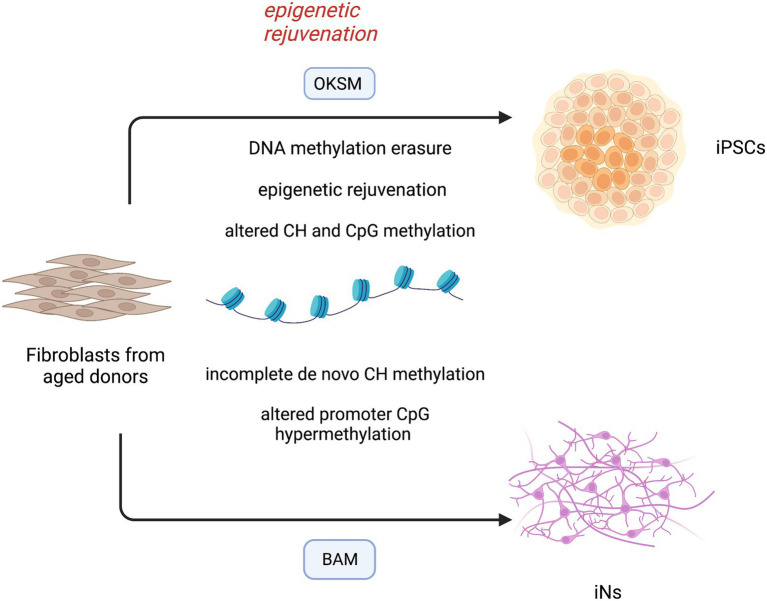
DNA methylation landscape in induced-pluripotent stem cells (iPSCs) and directly converted neurons (iNs). Reprogramming of aged fibroblasts to the pluripotent cell stage is characterized by methylation erasure and reset of epigenetic clock. The epigenetic rejuvenation occurs before complete dedifferentiation to iPSCs, suggesting novel treatments for age-associated diseases. During this process, a large reconfiguration of DNA methylation leads to aberrant CH and CpG methylation in differentiated cells. iNs derived from aged fibroblasts bypass the intermediate pluripotent cell stage, but still lack a faithful CH DNA methylation and show an altered CpG hypermethylation pattern at promoter regions. OKSM (OCT4, KLF4, SOX2, C-MYC); BAM (ASCL1, BRN2, MYT1L).

Importantly, human neurons have a much more extended developmental timeline compared to mouse neurons and reach the maximal *in vivo* mCH methylation in late adolescence (Lister et al., 2013). Therefore, an equal report that compares mCH methylation in ESC-derived human neurons and *in vivo* neurons is compelling and needs to be assessed in adult brain tissues. DNA methylation patterns of iPSCs and iNs have been recapitulated in Figure 2.

### Mitochondrial function

Epigenetic changes may contribute to many of the hallmarks of aging, including mitochondrial dysfunction (Booth and Brunet, 2016). Mitochondria are a major generator of energy in our cells and a major target of cellular aging and age-related NDDs (Booth and Brunet, 2016; Mertens et al., 2018; Gudenschwager et al., 2021). In neurons, these organelles play a fundamental role in synaptic transmission and cell survival, by regulating energy metabolism, cell death, reactive oxygen species (ROS) production, calcium homeostasis, and macromolecule biosynthesis. Neurons rely almost completely on mitochondria-mediated oxidative phosphorylation of glucose to fulfill their energy requirements and a major part of this energy is used to maintain the electrochemical gradient across the plasma membrane which, in turn, is necessary for the synaptic transmission. Also, mitochondria contribute to homeostasis of calcium which is involved in neurotransmitters release and signal transmission. All these functions are accompanied by a modest increase in ROS which act as signaling molecules at physiological range but cause oxidative stress and cell death at higher concentrations. As cell ages, mitochondrial turnover declines, leading to alteration in glucose metabolism, increase in ROS and mutations in mitochondrial DNA, all contributing to neurodegeneration (Booth and Brunet, 2016; Mertens et al., 2018; Gudenschwager et al., 2021). Hence, mitochondrial function is another aspect that must be assessed in models of neurons derived from iPSCs and directly induced from fibroblasts. Suhr and colleagues firstly examined the properties of mitochondria in fibroblast-derived iPSCs and in fibroblasts re-derived from iPSCs, finding a dramatic improvement of mitochondrial quality and function in both cells (Suhr et al., 2010). iPSCs were induced from aged fibroblasts, indicating that iPSCs reset the mitochondrial age to an embryonic-like state. Noteworthy, fibroblasts re-differentiated from iPSCs show an improvement in mitochondrial functionality with respect to both iPSCs and parental fibroblasts lines (Suhr et al., 2010). This result was confirmed by another study showing that the induction of pluripotency can reset the metabolic age of senescent and centenarian cells (Lapasset et al., 2011). Cellular reprogramming to the pluripotent state has also proven to rejuvenate the mitochondrial-associated cell death mechanisms to an embryo stage, even from starting fibroblasts that harbor chromosomal aberrations (Prigione et al., 2011). These cells restore mitochondrial function and the mitochondrial-mediated apoptotic signaling, crucial to prevent the oncogenic transformation that may occur in presence of chromosomal and genomic mutations. Despite being promising for the safety profile, these data suggest that iPSCs may be not ideal to study the organellar dysfunction in age-related neurodegeneration.

Instead, a study conducted by Kim and colleagues showed that iNs exhibit mitochondrial aging defects (Kim et al., 2018). Neurons directly induced from old fibroblasts show decreased expression of oxidative phosphorylation genes and impaired mitochondrial function, as assessed by increased mitochondrial fragmentation and lower total ATP levels with respect to neurons derived from young fibroblasts. As expected by previous results, iPSC-derived neurons from aged donors show no age-related mitochondrial defects, extending the mitochondrial rejuvenation that follows iPSC induction to neuronal differentiation (Kim et al., 2018).

Surprisingly, although old fibroblast-derived iNs show severe mitochondrial defects, their parental fibroblasts show only mild age-related phenotypes (Kim et al., 2018), proposing that upon neuronal transdifferentiation, iNs acquire a neuronal-specific age-dependent mitochondrial dysfunction, rather than preserving a general age-related mitochondrial status. This aspect further validates iNs as a valuable cell source for the study of age-related bioenergetic dysfunctions directly in human neurons and emphasizes the cell-type specificity of the aging process.

### Telomeres

Telomeres are special regions of repeated nucleotide sequences at the end of the chromosomes. Associated with specific proteins, they ensure the protection of the chromosomal ends from progressive degradation occurring during replication and prevent DNA repair systems from mistaking the very ends of the DNA strand for a double-strand break. The chance of the latter increases as the average telomere length decreases. The average telomere length is set and maintained in cells of the germline which typically express high levels of telomerase, whereas in somatic cells, telomere length is very heterogeneous but typically declines with age. Thus, telomere length has emerged as an indicator of replicative senescence and advancement of aging process (Aubert and Lansdorp, 2008). Importantly, the rate of increase in the percentage of short telomeres over time rather than the absolute telomere length has emerged as predictor of lifespan in mice (Vera et al., 2012). The role of this factor in replicative aging of somatic cells is evident; however, its contribution to the aging process of post-mitotic cells, such as neurons, remains obscure. Despite not being an accurate age-predictor, telomere shortening was found to be related to neurodegeneration (Martin-Ruiz et al., 2006; Watfa et al., 2011; Kume et al., 2012). Vera and colleagues showed that the pharmacological downregulation of telomerase and following telomere shortening results in disease-specific phenotypes in human iPSC-derived DA neurons (Vera et al., 2012). These cells exhibit a loss of tyrosine hydroxylase (TH) expression during differentiation, which is a characteristic feature of early PD.

Thus, manipulating telomere length may be a valuable strategy to model late-onset disease in human iPSC-derived lineages. Still, this represents only a feature of aging and how it reproduces the physiological and pathological aspects in aging and age-related diseases, respectively, needs to be further evaluated.

### Proteostasis

Proteostasis or “protein homeostasis” ensures a balanced proteome within the cells, by promoting the correct protein folding, trafficking and degradation mediated by proteasomes or lysosomes. Biological mechanisms underlying these functions are reported to become progressively impaired with age. In virtue of the post-mitotic nature of the neurons and their highly vulnerability to protein accumulation, these mechanisms are strongly related to the late-onset neurodegenerative process.

Although a direct and full comparison between iPSC-derived neurons and iNs with respect to the age-related decline in proteostasis has not been conducted, some evidence shows that iNs are somehow different in this aspect, as they were found to recapitulate different phenotypic manifestations associated with HD (Victor et al., 2018). Medium spiny neurons (MSNs) that were differentiated from iPSCs were reported to be free of the classical huntingtin (HTT) aggregates that characterize HD and to lack an overt cell death phenotype (Victor et al., 2018).

Instead, patients directly converted MSNs consistently exhibited mutant HTT (mHTT) aggregates, mHTT-dependent DNA damage, mitochondrial dysfunction, and spontaneous degeneration over time in culture. It is likely that directly reprogrammed subtype-specific neurons, as well as iNs, retain age-associated features of patients’ cells and these hallmarks finally result in a more reliable manifestation of the modeled disease. In addition, nuclear pore permeabilization is linked to the correct protein compartmentalization, including TFs and regulatory proteins, and has emerged as another factor impaired during the aging process (Mertens et al., 2015). Nuclear pores are composed of nucleoporins that control the flux of proteins, RNAs, and other information between the nucleus and the cytoplasm, acting as important regulator of gene transcription and global nuclear organization. With age increasing they get more permeable to cytoplasmic proteins entering the nucleus and are increasingly leaky for nuclear proteins, impacting the subcellular localization of proteins and the chromatin architecture and transcription. In part, the altered permeability may be a consequence of the low turnover of extremely long-lived nucleoporins, hence, an effect of age-associated damage of proteins.

Interestingly, a significant impairment in nucleocytoplasmic compartmentalization was observed in iNs derived from old donor cells compared to iNs from young and middle-aged donors, proposing an age-dependent phenomenon (Mertens et al., 2015). In contrast, iPSC-derived neurons show no detectable impairment in nucleocytoplasmic functionality in iNs converted from young, middle-aged, and old donors, indicating that iPSC rejuvenation can restore this function in old cells (Mertens et al., 2015). Taken together, these data demonstrate that iNs retain the principal aging signatures of the primary cell, and manifest neuronal-specific aging signs compared to neurons derived from iPSCs, confirming that they are an age-equivalent *in vitro* model for neuronal degeneration. Finally, the aging hallmarks can hamper the reprogramming process, but some evidence shows an equal efficient direct conversion for young and old fibroblasts (Mertens et al., 2015).

## Impact of aging signatures in brain disease models

iPSC-derived and directly converted neurons for disease modeling: impact on disease phenotype. Despite many of the NDD-causing mutations have been largely characterized, it still results very difficult to conduct studies of drug screening due to the lack of a model that correctly resemble the human neuronal physiology. The different complexity of human neurophysiology renders the use of animal models an unsuitable tool for these types of diseases as demonstrated from the unsuccessfully translation of drugs derived from animal screening to clinical application. For this reason, the pursuit for a more human resembling disease model remains a sensitive issue. Promising results have been reached in recent years by differentiating neurons *in vitro* from iPSCs or from somatic cells. The big advantage of differentiating neurons from iPSCs is related to an increased plasticity of undifferentiated cells that could be more efficiently induced toward a mature phenotype. However, iPSC-derived neurons phenotype is more similar to an embryonic stage and show a reset in the epigenetic aging so resulting not optimal for modeling diseases related to aging. To overcome this issue the best chance seems to be represented by the direct reprogramming. The first attempt of direct reprogramming toward neuronal lineage was accomplished in 2010 when the Wernig group successfully converted mouse fibroblasts into iNs by expressing three TFs, namely BRN2, ASCL1, and MYT1L (Vierbuchen et al., 2010). Since then, many efforts have been applied to directly reprogram somatic cells to specific neuron subtypes. Most of these protocols are based on the co-expression of neuron subtype-specific TFs together with BAM factors. By using this approach functional induced DA neurons (iDANs) expressing TH and other midbrain markers have been generated from different somatic cells (Addis et al., 2011; Caiazzo et al., 2011; Pfisterer et al., 2011; Kim et al., 2012; Liu et al., 2012; Torper et al., 2013; Jiang et al., 2015; Park et al., 2015; Di Val et al., 2017; Yoo et al., 2017).

However, the conversion efficiency of iNs is relatively low, ranging from 5 to 30% depending on the starting cell type. To overcome this limit many protocols have been identified based on a semi-direct approach in which pluripotency factors for direct reprogramming (PDR) can generate expandable neuronal progenitor cells (iNPCs) that can be further differentiated into desired cells (Kim J. et al., 2011; Lu et al., 2013; Zhu et al., 2014; Lee et al., 2015). Still, the usage of pluripotency factors may alter the epigenetic and transcriptional aging signatures. The direct reprogramming from blood cells by using SOX2 and C-MYC has been proven to cause a loss of age-related DNA methylation signatures at the early stages of induced neuronal stem cells (iNSCs) which further erode across extended passaging, eventually resembling the methylation profile of iPSC-derived neuronal precursors. Moreover, the epigenetic resetting is accompanied by a lack of age-associated transcriptional signatures and absence of cellular aging hallmarks. iNSCs derived from single colonies display differences in methylation signature suggesting that during the SOX2-dependent direct reprogramming each iNSC undergoes an independent epigenetic reprogramming process (Sheng et al., 2018). Here, we summarize the most relevant updates in neurodegenerative disease modeling by using the iPSC or direct reprogramming strategies (Table 1).

**Table 1:** Disease-associated phenotypes in iPSC-derived and direct reprogramming-derived neurons.

Disease	Study	Strategy	Disease-associated phenotype
*PD*	Nguyen et al. (2011) Sánchez-Danés et al. (2012)	iPSC	α-synuclein aggregates ↑ oxidative stress genes ↓ number of neurites
	Seibler et al. (2011)	iPSC	↑ mitochondrial stress
	Miller et al. (2013)	iPSC + progerin	abnormal nuclear morphology DNA damage ↑ ROS dendritic degeneration ↓ TH expression ↑ Lewy body
	Vera et al. (2016)	iPSC + telomerase inhibitor	DNA damage ↑ ROS ↓ dendrites
	Lee et al. (2019)	Direct reprograming	Proteasomal stress ↑ cell death
*AD*	Yagi et al. (2011)	iPSC	↑ Aβ42
	Israel et al. (2012) Ochalek et al. (2017)	iPSC	↑ Aβ42 Tau phosphorylation GSK-3β activation
	Mertens et al. (2021)	Direct reprogramming	↑ immature neuronal signaling cell cycle re-entry ↑ ROS DNA damage aneuploid DNA contents
	Hu et al. (2015)	Direct reprogramming	↑ Aβ42 ↑ p-tau e tau
*ALS*	Chen et al. (2014)	iPSC	NF aggregation neurite swelling axonal degeneration
	Tang et al. (2017)	Direct reprogramming	DNA damage ↓ nuclear organization
	Liu et al. (2016)	Direct reprogramming	soma shrinkage hypoactivity ↓ action potential inability to form NMJs
*HD*	Jeon et al. (2012)	iPSC	Absent or slow protracted HTT aggregates
	Camnasio et al. (2012)	iPSC	no evident HD phenotype
	Liu et al. (2014a,b)	Direct reprogramming	HTT aggregation abnormal neurite outgrowth and branching
	Victor et al. (2018)	Direct reprogramming	HTT aggregates mitochondrial dysfunction
	Pircs et al. (2022)	Direct reprogramming	spontaneous degeneration decline in proteostasis Increased DNA methylation autophagic impairment

### Parkinson’s disease

PD is a neurodegenerative disorder caused by the progressive loss of DANs in the substantia nigra which manifests with alteration in motility as bradykinesia, resting tremor, rigidity, flexed posture, “freezing,” and loss of postural reflexes (Lees et al., 2009). The generation of DANs from iPSC has allowed the investigation of genetic mutations in the pathogenesis of the disease and has given a proof-of-concept of the beneficial effect of reverting such mutations. Several groups have successfully obtained DANs with specific mutation related to PD starting from patient-specific iPSCs (Nguyen et al., 2011; Seibler et al., 2011; Sánchez-Danés et al., 2012).

These DANs show some of the most common features of PD such as α-synuclein (SNCA) aggregates, overexpression of oxidative stress genes, lower number of neurites, caspase-3 activation (Nguyen et al., 2011; Sánchez-Danés et al., 2012), and upregulation of PGC-1a (Seibler et al., 2011). However, the passage through the state of undifferentiated cells causes the loss of age-related phenotypes, and to overcome this issue, several groups are trying to manipulate iPSC-derived DANs to restore the aging. Lorenz Studer’s group showed a restoration of age identity by overexpressing progerin, a truncated form of lamin A known to be associated with premature aging (Dechat et al., 2008). In iPSC-derived DANs, the overexpression of progerin results in aging-associated phenotypes as abnormal nuclear morphology, DNA damage, and ROS accumulation and more specific PD features as dendritic degeneration, TH expression loss, and Lewy body accumulation (Miller et al., 2013). Later, the same research group adopted another approach which employs the pharmacological inhibition of telomerase starting from the observation that during the process of mDAN differentiation there is a shortening of telomeres.

The treatment with this inhibitor does not interfere with DAN differentiation efficacy but results in neurons with shorter telomeres that mirrors the neuronal aging phenotype (Vera et al., 2016).

In 2015, the analysis of epigenome of iPSC-derived DANs from PD patients has highlighted the role of epigenetic modifications for the development of both monogenic and sporadic disease (Fernández-Santiago et al., 2015). They found that iPSC-derived DANs from PD patients present a DNA methylation enrichment in enhancers elements resulting in downregulation of TFs as FOXA1, NR3C1, HNF4, and FOSL2 which were already associated with the specification of substantia nigra (Ziller et al., 2013). They also found that the alteration of DNA methylation was absent in parental skin cells or iPSC and only reveal upon differentiation into DANs. The PD DAN DNA methylation profile resembles the one of neuronal culture not- enriched-in-DAN indicating a failure to fully acquire the epigenetic identity during the reprogramming (Fernández-Santiago et al., 2015). Kim’s group was able to generate human iNPCs starting from patients’ fibroblasts with familiar LRRK2-associated and sporadic PD. The cells so generated showed a normal ploidy and expressed neuronal precursors markers as N-CAD, PAX6, PLZF, and ZO1 and more mature ones during the differentiation (TH, MAP2, NEUN, SYNAPSIN2). Most importantly, the neurons generated also mimic the pathological features of PD neurons as shown by an enhanced susceptibility to proteasome stress induced by proteasome inhibition treatment which results in increased apoptosis measured both by cell viability and caspase 3 cleavage (Lee et al., 2019). PD patient-derived human iNPCs of Kim’s group also have been used to evaluate the therapeutic efficacy of cryptotanshinone (Lee et al., 2020), a drug that was already reported to prevent oxidative stress injury in 1-methyl-4-phenyl-1,2,3,6-tetrahydropyridine (MPTP)-induced mouse PD models (Yun et al., 2018). More recently, a breakthrough was made by Drouin-Ouellet et al., who generated functional iDANs from patients with idiopathic PD, showing specific age and PD-related impairments (Drouin-Ouellet et al., 2022).

They focused on autophagic impairments since its functions decrease with age and they have been previously implicated in PD pathophysiology. Both an alteration in baseline chaperone-mediated autophagy and stress-induced autophagy were demonstrated to be present in idiopathic PD-derived iDANs. Importantly, the altered response to starvation was specific to iDANs and was not observed in any other healthy or PD iNs.

To assess how the age-associated properties of the human donors affect PD-related pathology, the Drouin-Ouellet’s group asked whether the accumulation of lysosomal structures in healthy and PD-iNs was associated with the age of the donor (Drouin-Ouellet et al., 2022). In fact, they found a positive correlation between the age and the accumulation of lysosomes in neurites and a tendency toward a positive correlation of the accumulation with age of onset at diagnosis. Moreover, alterations in stress-induced autophagy observed in iNs from idiopathic PD patients led to changes in the levels of phosphorylated SNCA at the serine 129 site, whose accumulation is a hallmark of PD pathology that has been recapitulated for the first time in idiopathic PD. By contrast, any pSer129 SNCA puncta were detected in the resulting iPSC-iNs, supporting that the maintenance of age in iNs is crucial for modeling SNCA pathology in idiopathic forms of PD. Finally, direct reprogramming of DANs that maintain age-related signatures provides a faithful tool to study idiopathic PD (Drouin-Ouellet et al., 2017).

### Alzheimer’s disease

AD is the most common neurodegeneration in the elderly. The hallmarks of the AD are the accumulation of amyloid beta (Aβ) plaques, intracellular neurofibrillary tangles of hyperphosphorylated tau, and loss of synaptic connections which result in neuronal death. The disease phenotype is manifested by cognitive decline and behavior changes with memory impairment, language disturbance, and mood swings. Several genetic mutations in amyloid precursor protein (APP), presenilin 1/2 (PS1/2) and apolipoprotein E (APOE) have been associated with pathogenesis of familial AD (fAD). However, genetic cases represent the minority of the cases as most of AD develop as sporadic (sAD) without a clear genetic etiology with risk increasing exponentially with age (Deture and Dickson, 2019) Several groups have reported the successful generation of iPSC-derived neurons from AD patients’ fibroblasts (Yagi et al., 2011; Yahata et al., 2011; Israel et al., 2012; Kondo et al., 2013; Mahairaki et al., 2014; Muratore et al., 2014; Sposito et al., 2015; Ochalek et al., 2017).

Yagi and colleagues pioneered the *in vitro* modeling of AD by generating neurons from patients with PS1/2 mutation. These neurons display increased levels of amyloid β42 (Aβ42) secretion (Yagi et al., 2011). Later, it was described the generation of neurons with APP mutation from fAD (Israel et al., 2012) and from sAD (Ochalek et al., 2017) patients’ fibroblasts which similarly showed higher levels of Aβ42 followed by τ phosphorylation and GSK-3β activation.

Another important application of iPSC-derived AD neurons was found in drug screening. Their usage has been very useful in deeper understanding of the potential benefits of γ- and β-secretase inhibitors on Aβ accumulation (Yahata et al., 2011; Israel et al., 2012; Kondo et al., 2013; Mahairaki et al., 2014; Muratore et al., 2014).

However, iPSC-dependent AD modeling retains an important limitation as they mostly express the fetal 3R isoform of tau protein. The lack of mature tau could deeply affect the results obtained in terms of reversion of pathological phenotype and drug screening (Sposito et al., 2015). Modeling of AD was also accomplished by direct reprogramming fibroblasts of both fAD and sAD patients.

Fred Gage’s group was able to generate sAD iNs resembling the physiopathological characteristics of the disease with decrease of synapsis and reduction of neuronal functionality. The iNs mirror the gene expression of AD brains with the activation of immature neuronal signaling patterns and induction of cell cycle re-entry (YAP/TAZ, Notch, HIF1a, Nf-kB, c-Myc, p53, and TGF-b). Furthermore, AD iNs display elevated ROS, DNA damage, and aneuploid DNA contents which are responsible of inducing the undifferentiated state of neurons which is an important hallmark of the early stage of AD (Mertens et al., 2021).

Another seminal approach in generating AD iNs by direct reprogramming was described by Gang Pei’s group. They were able to produce chemically induced neuronal cells (chiNs) from human fibroblasts only by using a cocktail of seven small molecules and reach the same results of iPSC-derived iNs in terms of morphology, gene expression, and electrophysiology. They accomplished the direct chemical conversion of fibroblasts derived from fAD patients with APP and PS1 mutations. iNs so generated displayed accumulation of Aβ42 levels in two out of four patient-derived iNs and an accumulation of both phosphorylated and total level of tau in one out of four (Hu et al., 2015).

### Amyotrophic lateral sclerosis

ALS is a fatal adult-onset neurodegenerative disease characterized by progressive degeneration of motor neurons (MNs), with approximately 10% of all cases being familial. A multitude of ALS genes was found to be related to the autophagic system, including *P62*, *OPTN*, *VCP*, *UBQLN2* and *TBK*. The remaining 90% of ALS cases are classified as sporadic disease. For these patients, results from family aggregation studies have identified an overlap between ALS and common neurodegenerative disorders, including AD and PD, suggesting the existence of susceptibility genes that might increase the overall risk of neurodegeneration among relatives.

However, attempts to establish the complex genetic basis for sporadic ALS by identifying susceptibility genes have had little success. Apart from genetic susceptibility, cellular aging process takes pivotal roles in the development of ALS, but the mechanisms leading to selective MN loss around the age of onset remain poorly understood (Robberecht and Philips, 2013). This is largely due to the difficulty to obtain patient MNs in order to faithfully recapitulate patient-specific phenotype. Moreover, there is no current approved treatment for the disease, and therapeutics developed with model animals even if proven successful in ALS animals, have failed when translated to clinical trials (Chen et al., 2014). This poses the necessity to use human neurons to accurately recapitulate the specific human cellular physiology and the age-related characteristics of patients’ cells, with the crucial aim of investigating ALS molecular mechanisms and to develop new screening platforms for effective therapeutics.

In this effort, researchers used the iPSC strategy to obtain MNs that harbor ALS mutations. Chen and colleagues generated iPSC lines from fibroblasts that carry mutations in Cu/Zn superoxide dismutase (*SOD1*) gene, the primary genetic cause identified in both familial and sporadic ALS cases (Chen et al., 2014). They managed to model human ALS beyond the interindividual variability and heterogeneity of the reprogrammed cell population. They used iPSCs from ALS patients with different mutations (D90A and A4V SOD1) and employed transcription activator-like effector nucleases (TALEN)-based homologous recombination to correct the D90A SOD1 mutation as well as to express the same mutation in human ESCs. In addition, they used an iPSC-based reprogramming system that simply adds three small molecules to the initial neuroepithelial differentiation protocol to generate MNs with 90% efficiency of generation overcoming the issue of heterogeneity and immaturity of disease target cells. By using these strategies, they could compare the mutants and controls under the same human genetic background and establish a cause-effect relationship between disease mutations and MN defects. SOD1 mutations emerged as leading cause of selective neurofilament (NF) misregulation in MNs but not in other neurons, leading to NF aggregation, neurite swelling, and axonal degeneration.

Despite representing a useful tool to investigate the molecular mechanisms underlying monogenic forms of ALS, iPSC approach is not suitable to model the late-onset phenotype. By reverting the donor cell age to an embryonic-like state, it is uncertain whether the defects identified in these young neurons resemble those of disease- stage degeneration in adult human patients. In this aspect, direct reprogramming approach appears to be a promising strategy to obtain mature and functional MNs that carry the age status apart from the genetic background of patient donor cells (Tang et al., 2017). Tang and colleagues efficiently generated MNs from iPSCs or directly induced from donor fibroblasts (iMNs) and made a comparative analysis of many aging-associated feature (Tang et al., 2017). They assessed aberrant nuclear morphology and DNA damage by the number of H2AX foci, and measured SA-β-Gal activity, nuclear and chromosome architectures by nuclear lamina-associated protein 2α (LAP2α), H3K9me3 and heterochromatin protein 1γ (HP1γ).

To minimize the potential complications due to methodological variances, the group developed a protocol consisting of the same cocktail of four TFs NSIL (NGN2, SOX11, ISL1, and LHX3) that could be employed to derive MNs both from iPSCs and directly from donor fibroblasts (Tang et al., 2017). Interestingly, they reported equal efficiency of derived neurons irrespective of derived from the iPSCs or donor fibroblasts. iMNs from old donor had a much higher number of cells containing H2AX foci, a dramatic increase in cells positive for SA-β-Gal, and concomitant reduced levels of markers associated with heterochromatin and nuclear organization (LAP2α, H3K9me3, HP1γ). Instead, no differences on aging-associated markers were detected between iPSC-MNs from different aged-cells.

Collectively, these data show that direct reprogramming to MNs preserves the principal aging-associated hallmarks of donor cells, whereas these are reset in MNs after passage through the pluripotent stage (Tang et al., 2017). The study of Liu et al. confirmed direct reprogramming of subtype-specific neurons is a valuable approach for disease modeling and drug screening (Liu et al., 2016). They defined an efficient method to directly obtain mature and functional MNs from adult human patients (hiMNs), which is based on viral transduction of NSIL factors, followed by administration of neuron-induction media containing the extrinsic factors forskolin, dorsomorphin, and basic fibroblast growth factor. These neurons exhibit the cytological and electrophysiological features of spinal MNs and form functional neuromuscular junctions (NMJs) with skeletal muscles. Noteworthy is that hiMNs converted from ALS-patient fibroblasts carrying FUS mutations show disease-specific morphological and functional defects. These were manifested through the soma shrinkage, the mislocalization of FUS protein in the cytosol, survival deficits, dramatic deficits in action potential firing, and the inability to form NMJs. Interestingly, comparing FUS levels and subcellular localization in fibroblasts obtained from both ALS patients and healthy controls failed to detect a significant difference, in sharp contrast to hiMNs, suggesting that upon motor neuronal differentiation, these cells acquire cell-type specific features. Together, the disease phenotypes observed in hiMNs matched the pathological features found in post-mortem tissues.

Finally, the study of Liu and colleagues suggested that patient-specific hiMNs can be employed for drug identification and validation (Liu et al., 2016). In a pilot screen of small molecules that promote the survival of ALS-hiMNs, Kenpaullone emerged as the best candidate drug which can greatly improve the morphology and the survival of patients’ hiMNs, by promoting outgrowth and branching of neuronal processes, and can restore motor neuron excitability.

### Huntington’s disease

HD is caused by expansion of CAG repeats in the first exon of the huntingtin (HTT) gene. Mutant HTT is widely expressed and believed to induce neurodegeneration through abnormal interactions with other proteins, leading to many cellular alterations and ultimately cell death (Zuccato et al., 2010). Striatal MSNs expressing dopamine- and cAMP-regulated phosphoprotein (DARPP-32) undergo the greatest degeneration.

As other NDDs, modeling HD has been challenging with animal models since significant differences between rodent and human cells and between non-neuronal cells and neurons exist. Thus, iPSC-derived neurons and iNs from patients with HD have provided precious insights into pathological mechanisms.

Jeon and colleagues obtained striatal MSNs and GABAergic neurons from iPSC-derived from patients with a juvenile form of HD (Jeon et al., 2012). Although the differentiation of MSNs was finely assessed by the immunostaining of lateral ganglionic eminence progenitors specific-markers (GSH-2 and DLX2) and MSNs marker DARPP-32, the concrete value of the reprogrammed cells for disease modeling is questionable. By using an antibody which selectively binds to the toxic N-terminal fragment of the mutant HTT (EM48), they examined whether iPSC-derived neurons develop huntingtin aggregates, a neuropathological hallmark of HD, after differentiation or intracerebral transplantation.

Surprisingly, no EM48-positive aggregates were found in cultured or grafted HD-iPSC-derived cells, presumably because of the reprogramming process to obtain iPSCs which might have affected the development of a cellular HD phenotype. An alternative explanation may be that HD-iPSCs show slow protracted huntingtin aggregate formation and such aggregates might develop at later stages of transplantation. To test this hypothesis, HD iPSC-neural precursor cells (NPC) grafted into the lateral ventricle of mice were analyzed for EM48 expression after longer time survival (at 33 weeks and 40 weeks), showing that huntingtin aggregation was evident at these stages. However, it should be considered that this model reproduces a juvenile form of disease characterized by 72 repeats and this result is not representative for modeling of late-onset forms caused by a lower number of repeats. In fact, both genetics and age contribute to HD pathology, considering that the CAG repeat expansion in HTT correlates with age of disease onset, and disease manifestation is more prevalent with increasing age, independent of CAG repeat length. iPSC-based strategies to obtain MSNs are certainly useful for HD forms in which the genetic factor is predominant, but these may lack precious information such as the age signature of donor cells and impact the disease phenotype observed especially for late-onset diseases. Microarray profiling of 14 iPSC lines from HD patients revealed gene expression patterns that distinguish early onset versus late-onset HD, and revealed that transcriptional changes specifically associated with HD pathogenesis were only present in lines carrying longer repeats (Mattis et al., 2012). Instead, pathways not previously associated with HD pathogenesis, such as changes in calcium signaling, showed effects specific to the 60-repeat range. These data suggest that iPSC-based technology may be unsuitable to reproduce all the features of late-onset HD carrying lower CAG repeats, and it is consistent with previous studies reporting only an enhanced lysosomal activity (Camnasio et al., 2012), but not an evident HD phenotype in neurons derived from iPSCs of HD patients.

By contrast, neuron-like cells directly converted from HD patient fibroblasts obtained through modulation of cell-lineage-specific TFs or RNA processing, were found to recapitulate the major aspects of neuropathological characteristics of HD, including mutant HTT aggregation, as assessed by immunostaining with EM48, increased cell death upon neural differentiation and abnormal neurite outgrowth and branching which is in accordance with a previous *in vivo* study in which abnormal dendritic arbors and increased dendritic branching in spiny striatal neurons were identified in post-mortem HD patients’ brain sections (Liu et al., 2014a). Consistently, another investigation reported mutant HTT aggregates, mutant HTT-dependent DNA damage, mitochondrial dysfunction, and spontaneous degeneration in MSNs directly derived from patients’ fibroblasts carrying CAG repeats lower than 50, as this range reflects most adult-onset cases (Victor et al., 2018). The same group demonstrated that iPSC-based protocol alters mutant HTT aggregation propensity. They derived HD-iPSCs from adult HD fibroblasts and differentiated these iPSCs back into embryonic fibroblast-like cells (HEFs). Upon direct conversion of HD-HEFs to MSNs little to no aggregated mutant HTT was detectable in these cells. More importantly, ubiquitin proteasome system (UPS), the main protein quality control machinery in the cell, was collapsed in HD-MSNs in comparison to embryonic MSNs, which retained the proteasome activity comparable to iPSCs. In addition, by comparing gene expression in young versus old fibroblasts and MSNs, fibroblasts did not display drastic changes in the expression of UPS-related genes with age, but MSNs from older individuals showed a dramatic increase in the number of downregulated UPS-related genes (Victor et al., 2018). The data suggest that the proteostasis collapse in adult MSNs, but not in originating fibroblasts or iPSC-derived neurons, is dependent on the cellular age of converted neurons (Victor et al., 2018). Therefore, age retention is crucial for HD modeling since the age-associated decline in proteostasis, which in turn reflects the downstream mutant HTT aggregates, is absent in iPSC-derived neurons. Eventually, mutant HTT aggregation is responsible for other HD-associated hallmarks, including increased DNA damage followed by spontaneous degeneration.

More direct evidence on the contribution of aging in HD phenotype manifestation was then provided by investigating the properties of MSNs reprogrammed from HD-fibroblasts sampled before the disease onset Pre-HD-MSNs. These neurons appeared less vulnerable to mHTT-induced toxicity, with lower levels of cell death and oxidative DNA damage, even though they still contained mutant HTT aggregates at a similar level as symptomatic HD-MSNs (Victor et al., 2018).

Therefore, directly converted HD-MSNs provide a human cellular model more suitable for examining the contribution of age and genetic factors to late-onset diseases. DARPP32^+^ neurons derived from adult-onset HD patients with clinically relevant CAG repeat lengths (41Q–57Q) showed a direct relationship between BDNF protein expression, CAG repeat length, and disease onset (Monk et al., 2021). BDNF plays a key role in the differentiation and maturation of striatal MSNs, with reduced BDNF signaling strongly implicated in HD neuropathogenesis. The protein expression negatively correlated to CAG repeat length and positively correlated to age of symptoms onset at days 30 and 45 of differentiation. Thus, at the individual patient level, BDNF levels directly relate to key HD pathological features, which can be modeled using direct-to-iNP reprogramming. Another age-associated hallmark in HD is the increase in epigenetic aging rates, which was previously described in post-mortem brain tissues (Steve Horvath et al., 2016). Since directly converted neurons from HD patients retain the age of donor cell (Pircs et al., 2022), this factor was investigated in this neuronal model. Pircs and colleagues examined the epigenetic age and confirmed a significantly increased DNA methylation predicted biological age in neurons induced from HD patients with respect to the control neurons (Pircs et al., 2022).

Interestingly, this model helped to unveil many of the molecular mechanisms underlying autophagic impairment, a factor widely associated with HD pathogenesis, and pointed to a combination of age-related epigenetic alterations and mutant HTT-mediated post transcriptional processes as a potential explanation for these defects.

Exactly how aging and the epigenetic alterations impact the disease pathology and autophagy impairments is currently unknown, but this model will allow further investigation of mechanistic links between these phenomena.

## Current achievements and prospects

**Figure 3:**
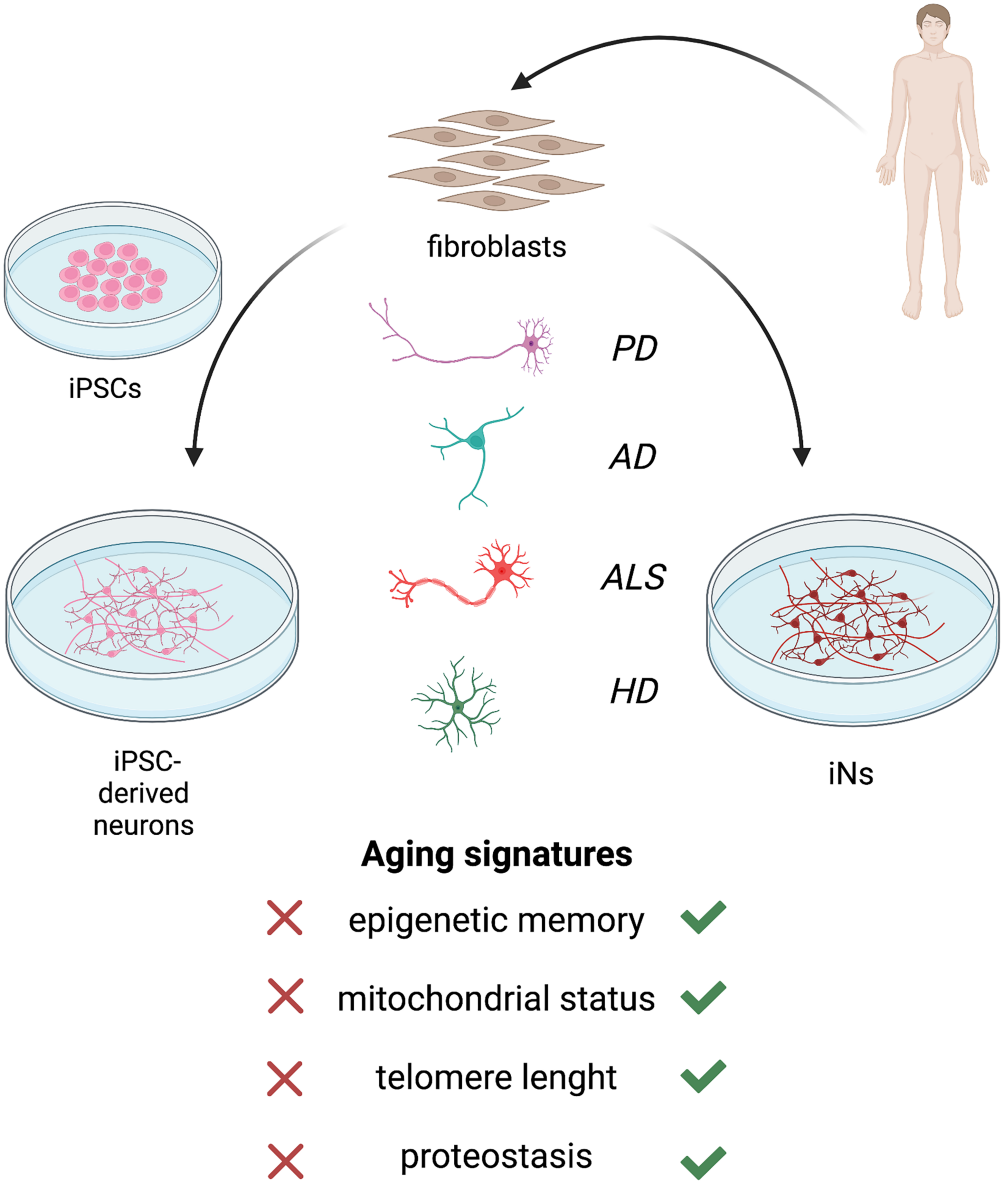
Patients’ fibroblast can be converted into neurons *via* induced pluripotent stem cell (iPSC)-based reprogramming **(left)** or direct reprogramming **(right)**. Both the approaches allow to obtain human neuronal models of neurodegenerative diseases, including Parkinson’s disease (PD), Alzheimer’s disease (AD), amyotrophic lateral sclerosis (ALS) and Huntington’s disease (HD). However, neurons and neuronal subtypes derived from iPSCs do not retain the main aging hallmarks of donor fibroblasts which are required for a reliable model of late-onset neurodegeneration. The induction of pluripotency reverts the cell age to an embryonic-like state, resetting epigenetic age, mitochondrial function, telomeres length, and proteostasis. Instead, direct reprogramming of induced neurons (iNs) bypasses the pluripotent intermediate state and retains the age of patients’ fibroblasts.

Aging hallmarks represent important contributors to neurodegeneration development and each aging feature differentially interacts with genetic and environmental factors in disease progression and manifestation. Also, its effect is related to the subtype-specific cell type/neuron (Kim et al., 2018; Victor et al., 2018; Pircs et al., 2022), underpinning the distinct involvement of aging in different NDDs. The relevance of age for late-onset NDDs development has been pictorially illustrated in Figure 3. Modeling neurodegeneration has been challenging due to the difficult accessibility in a source of human cells that could serve as a platform to study molecular mechanisms and test pharmacological treatments.

In this scenario, iPSC differentiation and direct neural conversion methods have emerged as promising and relatively reliable tools to obtain neurons that can recapitulate human disease phenotypes *in vitro*. However, iPSC-based technologies were reported to lack the aging signature of donor cell lines and to revert the cellular age to an embryonic-like state. The induction of pluripotency before neural differentiation restores age-associated cellular features, including epigenetic age, mitochondrial functions, telomere length, and proteostasis (Maherali et al., 2007; Meissner et al., 2008; Lapasset et al., 2011; Mertens et al., 2015).

Cell rejuvenation occurring in iPSCs and iPSC-derived neurons limits their use for disease modeling, since in some cases, they lack an overt disease-associated phenotype (Zhang et al., 2011; Camnasio et al., 2012; Mattis et al., 2012), whereas in other investigations, it is still uncertain whether the defects identified in young neurons resemble those of disease-stage degeneretion in adult human patients.

Neurons resemble those of disease-stage degeneration in adult human patients (Chen et al., 2014). In the attempts of including aging features in iPSC-derived neurons to study late-onset diseases, some researchers used brief progerin exposure, a truncated form of lamin A involved in premature aging (Miller et al., 2013).

Despite observing several age- and PD-related phenotypes not seen in previous iPSC studies, the question of whether targeting pathways that trigger progeroid syndromes mimics pathological over physiological aging remains elusive.

A more physiological approach exploited telomere shortening, but only a preliminary disease-related feature such as TH loss was observed in PD-iPSC-derived neurons and a wider characterization of the effects on disease manifestation is required to confirm the validity of this approach (Vera et al., 2016).

Apart from NDD modeling, a new wave of studies has been performed in the field of iPSC-related rejuvenation, which may have the potential for application in cell replacement therapy of age-related diseases. Recent studies are focusing on the “transient reprogramming” strategy, in which OKSM factors are briefly expressed in order to restore epigenetic age while retaining starting cell identity, raising the possibility of inducing cell rejuvenation *in vivo* (Olova et al., 2019; Gill et al., 2022). Directly induced neurons appear the most suitable strategy to model late-onset NDDs. Retaining all the main aging hallmarks of primary fibroblasts, iNs have proven to recapitulate age-related features in PD models (Lee et al., 2019, 2020; Drouin-Ouellet et al., 2022), ALS (Liu et al., 2016), and HD (Liu et al., 2014b; Victor et al., 2018; Monk et al., 2021; Pircs et al., 2022).

A major hurdle for the use of iNs for studying age-related diseases is the poor reproducibility and efficiency. The development of a platform to study NDDs and to find potential drugs requires an adequate number of reproducible cells. Translating the tool to a larger scale is challenging by using a direct neural conversion protocol, since it does not include a highly expandable intermediate stage and the neurons generated are postmitotic. The expandable phase of iN conversion depends entirely on the proliferation of fibroblasts, whereas the resultant yield of iNs strictly depends on the conversion rate. This also brings to a higher degree of variability in the converted cells due to the absence of clone selection. In contrast, iPSCs, once derived, can theoretically be expanded infinitely, making them suitable for the generation of large numbers of neurons for subsequent applications that require relatively large amounts of material, such as drug screening.

Direct conversion approach can be used from patients’ fibroblasts (Victor et al., 2018; Capano et al., 2022; Drouin-Ouellet et al., 2022; Pircs et al., 2022) and some evidence demonstrates that cells from all ages can be converted into iNs (Mertens et al., 2015). Noteworthy, a general difficulty in reaching equal conversion rates from adult or aged fibroblasts has been reported (Caiazzo et al., 2011; He et al., 2019), finally limiting the applicability of direct reprogramming for studying aging contribution in neurodegeneration. Improvements in conversion efficiencies especially from aged donors are needed before iN-based models can be translatable to large-scale investigations and pharmacological drug discovery in the treatment of age-related NDDs.

Interestingly, several studies are recently highlighting new molecular mechanisms involved in direct conversion process, therefore paving the way toward its optimization (Xie et al., 2018; Matsuda et al., 2019; Della Valle et al., 2020). Recently, Della Valle and colleagues proved that L1 retrotransposons are re-activated during the early stages of direct conversion in fully differentiated iDANs and inserted in specific regions of the genome relevant for neuronal lineage commitment and function (Della Valle et al., 2020). L1 retrotransposition is associated with increased chromatin accessibility and nearby lncRNA production in recipient loci. Moreover, blocking of L1 dynamics severely impairs the efficiency of iDAN transdifferentiation, suggesting that L1 retrotransposons are regulatory elements that elicit the expression of novel noncoding transcripts through chromatin remodeling. This confirms the role of both epigenetic mechanisms and non-coding RNAs (Pascale et al., 2022) in direct neural reprogramming and paves the way for a better understanding of the epigenetic mechanisms and their mechanistic link with downstream activation of lineage-specific genetic programs which can improve direct cell reprogramming-based technologies for future applications.

## Author contributions

SA, CC, and MC contributed to conception and design of the article. CC created the table. SA created the figure. SA and CC wrote the first draft of the manuscript. All authors contributed to the article and approved the submitted version.

## Funding

This work was supported by the H2020-FETOPEN-2018-2019-2020-01 ENLIGHT, Project number: 964497.

## Conflict of interest

The authors declare that the research was conducted in the absence of any commercial or financial relationships that could be construed as a potential conflict of interest.

## Publisher’s note

All claims expressed in this article are solely those of the authors and do not necessarily represent those of their affiliated organizations, or those of the publisher, the editors and the reviewers. Any product that may be evaluated in this article, or claim that may be made by its manufacturer, is not guaranteed or endorsed by the publisher.
